# Chronic Administration of Cannabinoid Receptor 2 Agonist (JWH-133) Increases Ectopic Ovarian Tumor Growth and Endocannabinoids (Anandamide and 2-Arachidonoyl Glycerol) Levels in Immunocompromised SCID Female Mice

**DOI:** 10.3389/fphar.2022.823132

**Published:** 2022-02-15

**Authors:** Henry L. Blanton, Melissa C. McHann, Haley De Selle, Canice Lei Dancel, Jose-Luis Redondo, Deborah Molehin, Nadezhda A. German, Scott Trasti, Kevin Pruitt, Isabel Castro-Piedras, Josée Guindon

**Affiliations:** ^1^ Department of Pharmacology and Neuroscience, Texas Tech University Health Sciences Center, Lubbock, TX, United States; ^2^ Department of Immunology and Molecular Microbiology, Texas Tech University Health Sciences Center, Lubbock, TX, United States; ^3^ Department of Pharmaceutical Sciences, Texas Tech University Health Sciences Center, Lubbock, TX, United States; ^4^ Department of Cell Biology and Biochemistry, Texas Tech University Health Sciences Center, Lubbock, TX, United States; ^5^ Center of Excellence for Translational Neuroscience and Therapeutics, Texas Tech University Health Sciences Center, Lubbock, TX, United States

**Keywords:** ovarian cancer, CB2 cannabinoid agonist, tumor growth, anandamide, JWH-133, 2-arachidnoylglycerol

## Abstract

Cannabinoid-based therapies are increasingly being used by cancer patients to treat chemotherapy-induced nausea and vomiting. Recently, cannabinoids have gained increased attention for their effects on cancer growth. Indeed, the effect of CB_2_ (JWH-015, JWH-133) agonists on breast cancer models have shown to reduce the size of breast cancer tumors. However, these studies assessing breast cancer progression were using CB_2_ agonist administered early into the cancer progression therefore assessing their effects on already established tumors is a critical need. In our study, we evaluate tumor growth using an ectopic xenograft ovarian (SKOV-3 and OVCAR-5) cancer model. The impact of chronic (30 days) administration of CB_2_ (JWH-133) agonist will be evaluated and started on 30 days of ectopic ovarian tumors. We will then evaluate and determine the mechanisms involved in ovarian cancer tumor growth by measuring levels of anandamide and 2-arachidonoyl glycerol as well as protein levels of CB_1_, CB_2_, ERα, ERβ, GPER, TNFα, IL-1β and IL-6 in ovarian and tumor tissues. Our results demonstrate a significant increase in ectopic ovarian tumor growth following chronic administration of JWH-133. Ovarian cancer tumor tissues chronically (30 days) treated with JWH-133 in comparison to vehicle treated groups showed an increase in endocannabinoid (AEA and 2-AG) and protein (CB_2_ and TNFα) levels with a decrease in GPER protein levels. Interestingly, our study emphasizes the importance of studying the impact of cannabinoid compounds on already established tumors to improve our understanding of cannabinoid-based therapies and, therefore better address clinical needs in cancer patients.

## 1 Introduction

One of the earliest known references to the use of cannabis for medicinal purposes is dated and appeared in a Chinese pharmacopoeia in 2800 BCE (Booth, 2003). Endocannabinoids are endogenous lipid-signaling molecules that mimic the pharmacological action of the principal psychoactive component of marijuana, delta-9-tetrahydrocannabinol (∆^9^-THC) (Gaoni and Mechoulam, 1964). Anandamide (AEA) (Devane et al., 1992) and 2-arachidonoyl glycerol (2-AG) (Mechoulam et al., 1995) are the two best-studied endocannabinoids. Endocannabinoids possess cannabimimetic properties because they bind and activate cannabinoid receptor 1 (CB_1_) (Devane et al., 1988) and/or cannabinoid receptor 2 (CB_2_) (Munro et al., 1993). AEA is mainly hydrolyzed by the fatty-acid amide hydrolase (FAAH) (Cravatt et al., 1996) whereas 2-AG is mainly, although not exclusively, hydrolyzed by the enzyme monoacylglycerol lipase (MAGL) (Goparaju et al., 1999; Dinh et al., 2002; Blankman et al., 2007). Indeed, the endocannabinoid system has emerged as a target for novel pharmacotherapies increasingly used by cancer patients to treat chemotherapy-induced nausea and vomiting (Parker et al., 2011; Kramer, 2015; Badowski, 2017). Clinical evidence shows that the active component of cannabis, ∆^9^-THC (Darkovska-Serafimovska et al., 2018; MacCallum and Russo, 2018) as well as pharmaceutical cannabinoids (Maida and Daeninck, 2016; Blake et al., 2017) can reduce chronic neuropathic pain in cancer patients. Recently, cannabinoids have gained increased attention for their effects on cancer growth. *In vitro* studies have shown that mixed CB_1_/CB_2_ agonist WIN55,212-2 reduces viability of human sarcoma cells (Luca et al., 2009) and demonstrates antiproliferative effects in human melanoma cells (Scuderi et al., 2011). Moreover, ∆^9^-THC has been shown to reduce the number of Ki67 immunoreactive nuclei, a cell cycle marker through the orphan cannabinoid receptor GPR55 (Kolbe et al., 2021). Cannabidiol has been demonstrated to enhance the inhibitory effects of ∆^9^-THC cell proliferation and survival of human glioblastoma cells (Marcu et al., 2010). Therefore, most of the studies investigating the effects of cannabinoid-based therapies on tumor growth have been using *in vitro* models that inadequately mimic *in vivo* cancer microenvironment and tumor progression (Guindon et al., 2011; Ladin et al., 2016). In our study, we decided to focus only on cannabinoid receptor 2 agonists since only a small number of preclinical studies have evaluated the impact of CB_2_ agonists and their effects following long-term treatment on tumor growth and cancer cell proliferation (Hanlon et al., 2016; Elbaz et al., 2017). Moreover, these studies evaluated the effects of CB_2_ agonists at an early stage of tumor growth (Hanlon et al., 2016; Zhang et al., 2018). Previous work has evaluated the effect of CB_2_ (JWH-015, JWH-133) agonists on breast cancer models (Hanlon et al., 2016; Elbaz et al., 2017; Zhang et al., 2018) and were able to reduce the size of breast cancer tumors. However, these studies assessing breast cancer progression were using CB_2_ agonist administered within 7 days of tumor transplant with small tumor volumes under 100 mm^3^ using peritumoral (Elbaz et al., 2017) or systemic (Hanlon et al., 2016) administration. Recent studies have shown that combination of JWH-133 and light inhibit tumor growth in triple negative breast cancer (Zhang et al., 2018), but once again the cannabinoid agonist 2 treatment is given at the beginning of tumor growth. There is an urgent need to improve our understanding of the long-term effects of cannabinoid-based therapies on tumor growth. Indeed, most studies have looked at the effect of cannabinoid compounds on tumor growth when the tumor starts growing. However, in cancer patients, the use of cannabinoid compounds is in the presence of already established and grown tumors. The possibility of enhanced tumor growth due to the side effects of analgesic compounds constitutes a significant problem that worsens the prognosis for cancer patients (Maley et al., 2017). Therefore, evaluating the effects of cannabinoid receptor 2 agonist in tumors that are already developed is critical. Indeed, to better understand the impact of cannabinoid receptor two (CB_2_) agonist on tumor growth, we will first evaluate tumor growth using an ectopic xenograft ovarian cancer model using two ovarian cancer cell lines (SKOV-3 and OVCAR-5). Then, we will have the ectopic xenograft ovarian cancer cell grow a tumor for 30 days before we evaluate the impact of chronic administration of CB_2_ (JWH-133) agonist for 30 days and starting injection of JWH-133 after 30 days of tumor growth. It has been demonstrated that the endocannabinoid system is involved in both the central and peripheral tissues (Ramer et al., 2019; Santoro et al., 2021) and that endocannabinoid (AEA, 2-AG) levels and their degrading enzyme (FAAH, MAGL) as well as cannabinoid receptor 1 (CB_1_) and 2 (CB_2_) are altered in sex-hormones dependent malignant tissues (Ramer et al., 2019; Santoro et al., 2021). Moreover, cannabinoid receptor 2 agonists have been shown to modulate inflammatory markers such as TNFα, IL-6 and IL-1β (Pan et al., 2020; Turcotte et al., 2015; Yu et al., 2019). Consequently, we will determine the mechanisms of action involved in ovarian cancer tumor growth, we will measure levels of anandamide and 2-arachidonoyl glycerol as well as protein levels of CB_1_, CB_2_, ERα, ERβ, GPER, TNFα, IL-1β and IL-6 in ovarian and tumor tissues. Hence, our study uncovers that increase in tumor growth following chronic administration of JWH-133 is associated with increased in endocannabinoid (AEA and 2-AG) and protein (CB_2_ and TNFα) levels in tumor tissues. Furthermore, this study addresses a critical point and emphasize the importance of studying the impact of cannabinoid compounds on already established tumors to improve translational aspect of preclinical studies and therefore, better address clinical needs in cancer patients.

## 2 Materials and Methods

### 2.1 Cells

The OVCAR-5 cancer cell line used in this manuscript was purchased from Cell Biolabs, INC. (San Diego, CA, United States) and the SKOV-3 cancer cell line is from ATCC (San Diego, CA, United States). These ovarian cancer cell lines were stored in liquid nitrogen upon arrival. Briefly, cells were thawed and expanded in McCoy’s 5A media, for OVCAR-5 cells the media was supplemented with 10% fetal bovine serum (FBS), 0.1 mM, MEM Non-Essential Amino Acids (NEAA), 2 mM L-glutamine, 1% Penicillin/Streptomycin antibiotics, and for SKOV-3 cells the media was supplemented with 10% Fetal Bovine Serum and 1% Penicillin/Streptomycin antibiotics. Cell lines were maintained in a humidified tissue culture incubator at 37^o^C and 5% C0_2_ (Maureen and Harrison, 1997) where they were not allowed to grow to confluency therefore subcultured at ∼80% confluence, and the medium was renewed three times per week.

### 2.2 Animals

Experiments were performed using six to 8 weeks old female immune-compromised SCID-SHO mice obtained from Charles River Laboratories (Wilmington, MA, United States), and maintained in a pathogen-free environment within the Laboratory Animal Resource Center (LARC). Mice were group housed (4 per cage) under a 12:12 h light-dark cycle (lights on 07:00, lights off 19:00) and provided with standard mouse chow containing 19.8% protein (Purina Lab Diet, St-Louis, MO, United States) and water *ad libitum*. All animal care and experimental procedures used in this study were approved by the Institutional Animal Care and Use Committee (IACUC) of the Texas Tech University Health Sciences Center and conducted in accordance with the National Institutes of Health (NIH) accepted guidelines found in the Guide for the Care and Use of Laboratory Animals (National Research Council DoEaLS, Institute for Laboratory Animal Research, 2011)

### 2.3 Drugs

JWH-133 was obtained from Cayman Chemicals (Ann Arbor, Michigan, United States). JWH-133 was stored at −20^o^C. Vehicle and JWH-133 (1 mg/kg) solutions were prepared fresh on the day of experiment. The vehicle solution for control experiments was composed of ethanol, DMSO, Tween80, and physiological saline prepared in a 1:1:1:17 ratio (5% ethanol: 5% DMSO: 5% Tween80: 85% physiological saline (0.9% NaCl)). JWH-133 was dissolved and administered in the same 1:1:1:17 vehicle solution. Vehicle and JWH-133 were administered intraperitoneal (i.p.) at a volume of 10 μl/g of body weight.

### 2.4 Xenograft Tumor Growth

**FIGURE 1:**
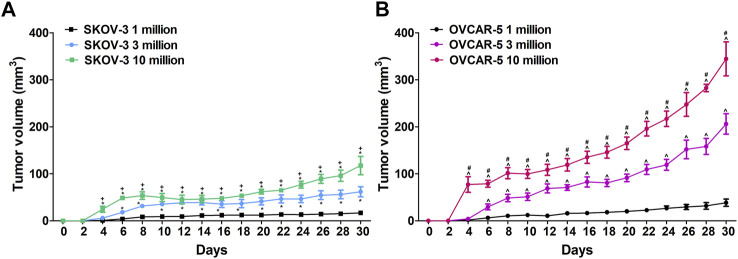
**(A)**Difference in ovarian SKOV-3 and OVCAR-5 cancer ectopic xenograft tumor growth following 1, 3 and 10 × 10^6^ cells subcutaneous administration in immunocompromised SCID-SHO female mice. **(B)**Increase in tumor growth was shown following administration of 10 million ovarian (OVCAR-5) cancer cells relative to 1 or 3 million in immunocompromised SCID-SHO female mice. Symbols represent the means ± SEM. (1 million, *n* = 8 for SKOV3, *n* = 11 for OVCAR5 in SCID-SHO females; 3 million, *n* = 6 for SKOV3, n = 9 for OVCAR5 in SCID-SHO females; 10 million, *n* = 8 for SKOV3, *n* = 10 for OVCAR5 in SCID-SHO females). *p < 0.037 for SKOV-3 10 or 3 million vs SKOV-3 1 million (repeated measures one-way ANOVA with Bonferroni post hoc); + *p* < 0.044 for SKOV-3 10 million vs SKOV-3 3 million (repeated measures one-way ANOVA with Bonferroni post hoc); ^ *p* < 0.02 for OVCAR-5 10 or 3 million vs OVCAR-5 1 million (repeated measures one-way ANOVA with Bonferroni post hoc; #*p* < 0.011 for OVCAR-5 10 million vs OVCAR-5 3 million (repeated measures one-way ANOVA with Bonferroni post hoc).

Ovarian cancer cells (OVCAR-5 and SKOV-3) used for mice injections were consistently obtained from passages 3 thru 5 to maintain homogeneity and to avoid genomic variations from further time in culture. The cell lines were plated 140 mm cell culture dishes and grown until 70–80% confluence. On the day of injection, cells were harvested after a brief incubation in trypsin-EDTA solution, counted using a hemocytometer, centrifuged and reconstituted in McCoy’s media with no supplements. Cells were prepared to be injected subcutaneously in immunocompromised SCID-SHO female mice at a concentration of 1, 3 or 10 million cells in 0.2 ml (see Figure 1).

OVCAR-5 and SKOV3 cells were injected subcutaneously (right flank) to immunocompromised SCID-SHO female mice at different concentrations (1, 3 or 10 million cells in 0.2 ml) and tumor growth was assessed by measuring every 2 days ectopic ovarian tumor growth using a digital caliper (formula (½ (a x b^2^); length (a), width (b); Dubey et al., 2015).

Thirty days after the injection of the cell line, vehicle and JWH-133 were administered intraperitoneal (i.p.) daily (systemic) at a volume of 10 μl/g of body weight from day 30 to day 60 and measurement of ectopic ovarian tumor growth every 3 days using a digital caliper (Dubey et al., 2015).

Mice were euthanized after 60 days of tumor growth and tissue samples (Ovaries and ectopic xenograft ovarian tumors) were rapidly dissected and fast frozen in 2-methylbutane precooled on dry ice (−30^o^C) and stored at −80^o^C until use as described previously (Guindon et al., 2011; Guindon et al., 2013).

### 2.5 LC/MS/MS analysis of Endocannabinoids (Anandamide and 2-Arachidonoyl Glycerol)

#### 2.5.1 Lipid Extraction

Lipids were extracted from ovaries and ovarian cancer tumors in the same method as described previously (Bradshaw et al., 2006; Guindon et al., 2013). In brief, frozen tissue was weighed and place in centrifuge tubes on ice. 40:1 volumes of methanol were added to each tube followed by 10 µl of 100pM deuterium-labeled N-arachidonoyl glycine (d8NAGly; Cayman Chemical, Ann Arbor, MI, United States) to act as an internal standard. Samples were then covered with parafilm and left on ice and in darkness approximately 2 h. Remaining on ice, samples were homogenized using a polytron for approximately 1 min. Samples were centrifuged at 19,000 x g at 24^O^C for 20 min. Supernatants were collected and placed in polypropylene tubes and HPLC-grade water was added making the final supernatant/water solution 25% organic. To isolate the compound of interest partial purification of the 25% solution was performed on a Preppy apparatus assembled with 500 mg C18 solid-phase extraction columns. The columns were conditioned with 5 ml of HPLC-grade methanol immediately followed by 2.5 mol of HPLC-grade water. The supernatant/water solution was then loaded onto the C18 column, and then washed with 2.5 ml of HPLC grade water followed by 1.5 ml of 40% methanol. Elutions of 1.5 ml of 70%, 85%, and 100% methanol were collected in individual autosampler vials and then stored in a −20°C freezer until mass spectrometer analysis.

#### 2.5.2 LC/MS/MS Quantification

Samples were removed from the −20°C freezer, allowed to warm to room temperature for 10 min, and vortexed for approximately 1 min before being placed into the autosampler, where they were held at 24°C (Agilent 1100 series autosampler, Palo Alto, CA) for LC/MS/MS analysis. 10–20 µl of eluants were injected separately for each sample to be rapidly separated using a C18 Zorbax reversed-phase analytical column to scan for each individual lipid. Gradient elution (200 µl/min) then occurred, under the pressure created by two Shimadzu 10AdVP pumps (Columbia, MD). Next, electrospray ionization was accomplished using an Applied Biosystems/MDS Sciex (Foster City, CA) API3000 triple quadrupole mass spectrometer. A multiple reaction monitoring (MRM) setting on the LC/MS/MS was then used to analyze levels of each lipid present in the sample injection. Synthetic standards were used to generate optimized MRM methods and standard curves for analysis.

#### 2.5.3 LC/MS/MS Analysis

The amount of analyte in each sample was calculated using a combination of calibration curves of the synthetic standards obtained from the Analyst software and recovery adjusted by the deuterium-labeled internal standard. The standards provided a reference for the retention times by which the analytes could be compared. They also helped to identify the specific precursor ion and fragment ion for each analyte which enabled their isolation. These processes provide confidence in the claim that the compounds measured were, in fact, the compound of interest. The amount of each compound in each tissue was then converted to ng per gram of tissue.

### 2.6 Western Blot analysis Measuring Proteins Levels of CB_1_, CB_2_, ERα, ERß, GPER, TNFα, IL-6 and IL-1ß

Western blots were performed as described previously (Castro-Piedras et al., 2021). Briefly, tumor tissue was homogenized directly in RIPA buffer supplemented with proteinase inhibitors (Thermo Scientific, Massachusetts, United States). Protein concentrations were determined using a BCA assay (Thermo Scientific, Massachusetts, United States). Lysates of equal protein concentrations were subjected to polyacrylamide gel electrophoresis using Invitrogen Bolt gel system, transferred to PVDF (Millipore, Massachusetts, United States) membranes, and immunoblotted. Antibodies used are as follows: CB_1_ (ab 23703) at 1: 500 dilution, CB_2_ (ab3561) at 1: 500 dilution, GPER (ab39742) at 1: 500 dilution, TNF alpha (ab215188) at 1:1000, ER alpha (CST #8644) at 1:1000 dilution, ER beta (ab3576) at 1:500 dilution, IL-6 (ab233706) at 1:1000 dilution, IL-1 beta (ab2105) at 1:1000 dilution, and GAPDH (Sc-47724) at 1:1000 dilution. Membranes were incubated in 5% milk/TBST with primary antibody overnight at 4°C. Membranes were washed with TBST and probed with horseradish peroxidase-conjugated secondary antibodies in 5% milk/TBST for 1 h at room temperature (RT). Membranes were visualized and imaged by enhanced chemiluminescence (ECL) reagent (Thermo Scientific) in Azure C300 gel imaging system (Azure Biosystems).

Densitometry measurements were performed using Image J software (National Institutes of Health, Bethesda, MD); GAPDH was used as a loading control.

### 2.7 Data analysis and Statistics

All experiments were conducted in a blinded manner. Animals were randomly assigned to experimental conditions. Tumor growth for each concentration (1, 3 or 10 million cells in 0.2°ml) or treatment (vehicle or JWH-133 1 mg/kg) groups were expressed as mean ± SEM. Data were analyzed using analysis of variance (ANOVA) for repeated measures with time as a factor or one-way or two-way ANOVA as appropriate. The Greenhouse-Geisser correction was applied to all repeated factors; degrees of freedom reported for significant interactions are the uncorrected values. The source of significant interactions was further evaluated by performing one-way ANOVAs at each individual time point, followed by Bonferroni post hoc tests. The different components of the total variation were settled *a priori* using multiple regression analysis (Draper and Smith, 1998). Effects of JWH-133 on endocannabinoid and protein levels in ovaries and tumors were analyzed using unpaired t-tests (one-tailed). Analyses were performed using SPSS statistical software (version 25.0, SPSS Incorporated, Chicago, IL, United States). *p* < 0.05 was considered significant.

## 3 Results

### 3.1 Difference in Ovarian SKOV-3 Cancer Ectopic Xenograft Tumor Growth Following 1, 3 and 10 × 10^6^ Cells Subcutaneous administration in Immunocompromised SCID-SHO Female Mice

In immunocompromised SCID-SHO female mice, significant differences between the groups (1, 3 and 10 million administration of SKOV-3 cancer cells) (F_2,19_ = 63.04, *p* < 0.0001) were found in a time- (F_14,266_ = 24.63, *p* < 0.0001) and time-group (F_28,266_ = 5.24, *p* < 0.0001) dependent manner (Figure 1A). Indeed, a significant increase in tumor growth was shown following administration of 10 million ovarian (SKOV-3) cancer cells relative to 3 million in immunocompromised SCID-SHO female mice from day 4 (*p* < 0.021) to day 8 (*p* < 0.044) and, from day 24 (*p* < 0.009) to day 30 (*p* < 0.031) (Figure 1A). Moreover, a significant increase in tumor growth was shown following administration of 10 million ovarian (SKOV-3) cancer cells relative to 1 million in immunocompromised SCID-SHO female mice from day 4 (*p* < 0.002) to day 30 (*p* < 0.0001) (Figure 1A). Furthermore, a significant increase in tumor growth was shown following administration of 3 million ovarian (SKOV-3) cancer cells relative to 1 million in immunocompromised SCID-SHO female mice from day 6 (*p* < 0.024) to day 28 (*p* < 0.016) (Figure 1A).

### 3.2 Difference in Ovarian OVCAR-5 Cancer Ectopic Xenograft Tumor Growth Following 1, 3 and 10 × 10^6^ Cells Subcutaneous administration in Immunocompromised SCID-SHO Female Mice

In immunocompromised SCID-SHO female mice, significant differences between the groups (1, 3 and 10 million administration of OVCAR-5 cancer cells) (F_2,27_ = 73.05, *p* < 0.0001) were found in a time- (F_14,378_ = 128.90, *p* < 0.0001) and time-group (F_28,378_ = 26.43, *p* < 0.0001) dependent manner (Figure 1B). Indeed, a significant increase in tumor growth was shown following administration of 10 million ovarian (OVCAR-5) cancer cells relative to 3 million in immunocompromised SCID-SHO female mice from day 4 (*p* < 0.0001) to day 30 (*p* < 0.002) (Figure 1B). Moreover, a significant increase in tumor growth was shown following administration of 10 million ovarian (OVCAR-5) cancer cells relative to 1 million in immunocompromised SCID-SHO female mice from day 4 (*p* < 0.0001) to day 30 (*p* < 0.0001) (Figure 1B). Furthermore, a significant increase in tumor growth was shown following administration of 3 million ovarian (OVCAR-5) cancer cells relative to 1 million in immunocompromised SCID-SHO female mice from day 6 (*p* < 0.02) to day 30 (*p* < 0.0001)(Figure 1B).

### 3.3 Increase in Ovarian (OVCAR-5) Cancer Ectopic Xenograft Tumor Growth Following Chronic administration of JWH-133 (1 mg/kg) in Immunocompromised SCID-SHO Female Mice

**FIGURE 2:**
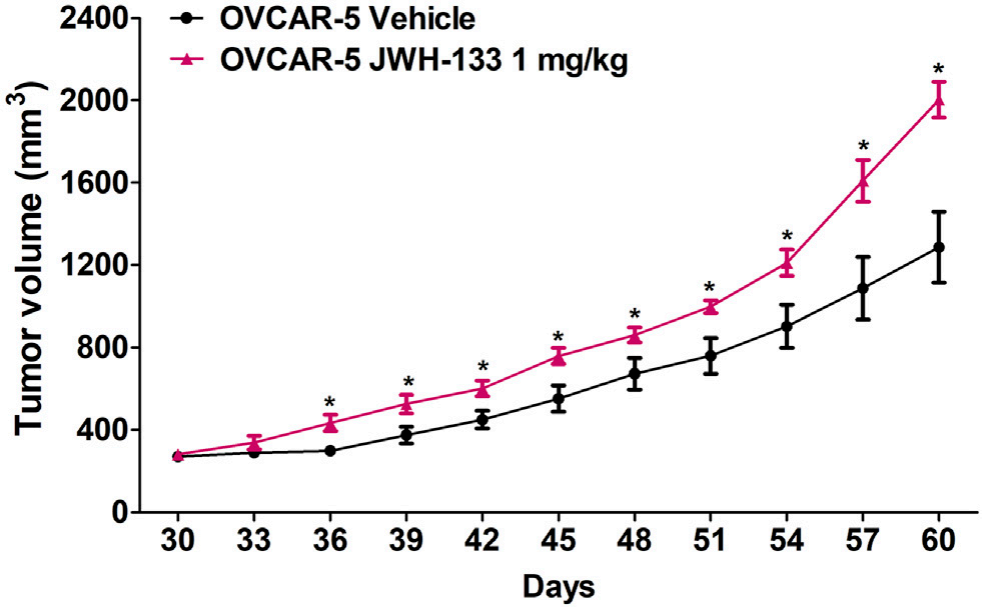
Increase in ovarian (OVCAR-5) cancer ectopic xenograft tumor growth following chronic daily administration of JWH-133 (1 mg/kg) relative to vehicle group in immunocompromised SCID-SHO female mice. JWH-133 (1 mg/kg i.p.) chronic daily administration for 30 days starting on day 30 of tumor growth produced an increase in OVCAR-5 ectopic xenograft ovarian cancer growth in immunocompromised SCID-SHO female mice relative to the vehicle treated group. Symbols represent the means ± SEM. (OVCAR-5 tumor growth in SCID-SHO female mice receiving vehicle, *n* = 8 SCID-SHO females; OVCAR-5 tumor growth in SCID-SHO female mice receiving JWH-133, *n* = 8 SCID-SHO females). *p < 0.044 for JWH-133 (1 mg/kg (repeated measures one-way ANOVA).

In immunocompromised SCID-SHO female mice, significant differences between the groups (daily administration of vehicle or JWH-133 1 mg/kg in OVCAR-5 cancer ectopic xenograft model) (F_1,14_ = 13.39, *p* < 0.003) were found in a time- (F_10,140_ = 113.37, *p* < 0.0001) and time-group (F_10,140_ = 6.19, *p* < 0.0001) dependent manner (Figure 2). Indeed, a significant increase in ectopic xenograft tumor growth was shown following chronic administration of JWH-133 (1 mg/kg i.p.) from day 36 (*p* < 0.014) to day 60 (*p* < 0.002) relative to vehicle group (Figure 2).

### 3.4 No Change in the Levels of 2-Arachidonoyl Glycerol and Anandamide Levels in the Ovaries of Control (No OVCAR-5 Tumor) in Immunocompromised SCID-SHO Female Mice

**FIGURE 3:**
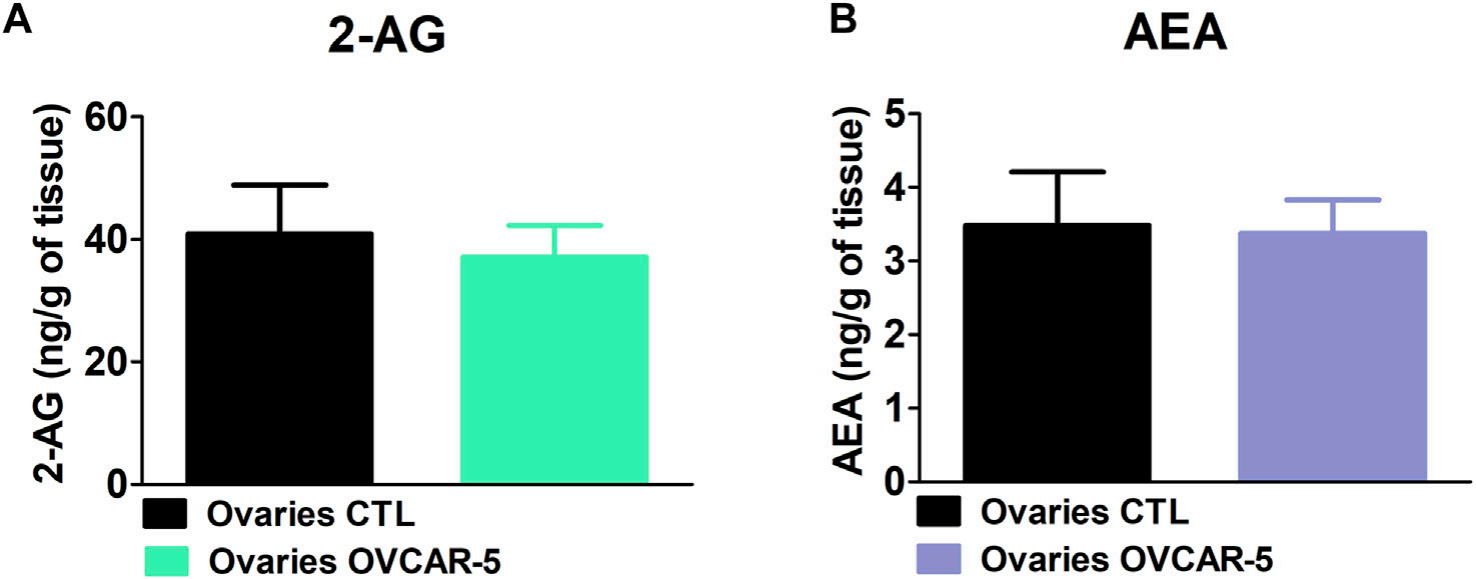
No change in the levels of 2-arachidonoyl glycerol (2-AG) and anandamide (AEA) levels in the ovaries of control (no OVCAR-5 tumor) relative to OVCAR-5 ectopic xenograft tumor in immunocompromised SCID-SHO female mice. No changes in AEA **(A)** and 2-AG **(B)** levels were observed in the ovaries of control (no OVCAR-5 ectopic tumor) in comparison to OVCAR-5 ectopic ovarian tumor in immunocompromised SCID-SHO female mice. Data are expressed as mean ± S.E.M. (ovaries in control (no OVCAR-5 tumor) SCID-SHO female mice, *n* = 5–7 SCID-SHO females; ovaries in OVCAR-5 tumor SCID-SHO female mice, *n* = 5–7 SCID-SHO females).

No changes in 2-AG levels were observed in the ovaries of control (no OVCAR-5 ectopic tumor) in comparison to OVCAR-5 ectopic tumor in SCID-SHO female mice (t_7_ = 0.334, *p* = 0.3741 (ns) unpaired t-test (one-tailed)) (Figure 3A). No changes in AEA levels were observed in the ovaries of control (no OVCAR-5 ectopic tumor) relative to OVCAR-5 ectopic tumor in SCID-SHO female mice (t_7_ = 0.380, *p* = 0.3570 (ns) unpaired t-test (one-tailed)) (Figure 3B).

### 3.5 Increase in 2-Arachidonoyl Glycerol Levels but not in Anandamide Levels in the Ovaries of SCID-SHO Female Mice With OVCAR-5 Ectopic Xenograft Tumors Receiving Chronic Systemic administration of JWH-133

**FIGURE 4:**
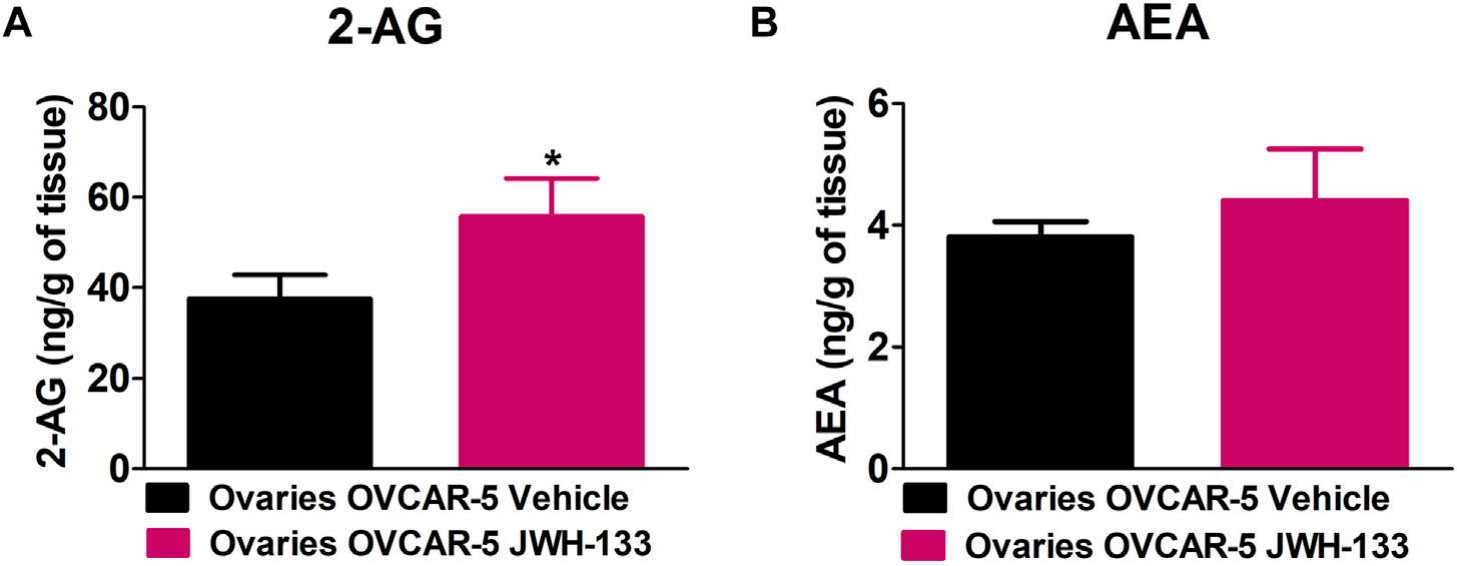
Increase in 2-arachidonoyl glycerol (2-AG) levels but not in anandamide (AEA) levels in the ovaries of SCID-SHO female mice with OVCAR-5 ectopic xenograft tumors receiving chronic systemic administration of JWH-133. JWH-133 (1 mg/kg i.p.) chronic administration for 30 days starting on day 30 of tumor growth produced an increase in 2-AG **(A)** levels in the ovaries of OVCAR-5 ectopic xenograft female mice relative to the vehicle treated group. No changes in AEA **(B)** levels were observed following chronic administration of JWH-133 relative to vehicle-treated group in the ovaries of OVCAR-5 ectopic xenograft female mice. Symbols represent the means ± SEM. (ovaries of OVCAR-5 tumor in SCID-SHO female mice receiving vehicle, *n* = 4-6 SCID-SHO females; ovaries of OVCAR-5 tumor in SCID-SHO female mice receiving JWH-133, *n* = 6 SCID-SHO females). *p < 0.0386 unpaired t-test (t_11_ = 1.950, one-tailed).

JWH-133 (1 mg/kg i.p.) chronic administration for 30 consecutive days starting on day 30 of tumor growth produced an increase in 2-AG levels in the ovaries of OVCAR-5 ectopic xenograft female mice relative to the vehicle treated group (t_11_ = 1.950, *p* < 0.0386 unpaired t-test (one-tailed)) (Figure 4A). No changes in AEA levels were observed following chronic administration of JWH-133 relative to vehicle-treated group in the ovaries of OVCAR-5 ectopic xenograft female mice (t_11_ = 1.105, *p* = 0.1464 (ns) unpaired t-test (one-tailed)) (Figure 4B).

### 3.6 Increase in 2-Arachidonoyl Glycerol and Anandamide Levels in the OVCAR-5 Ectopic Xenograft Tumors of SCID-SHO Female Mice Receiving Chronic Systemic administration of JWH-133

**FIGURE 5:**
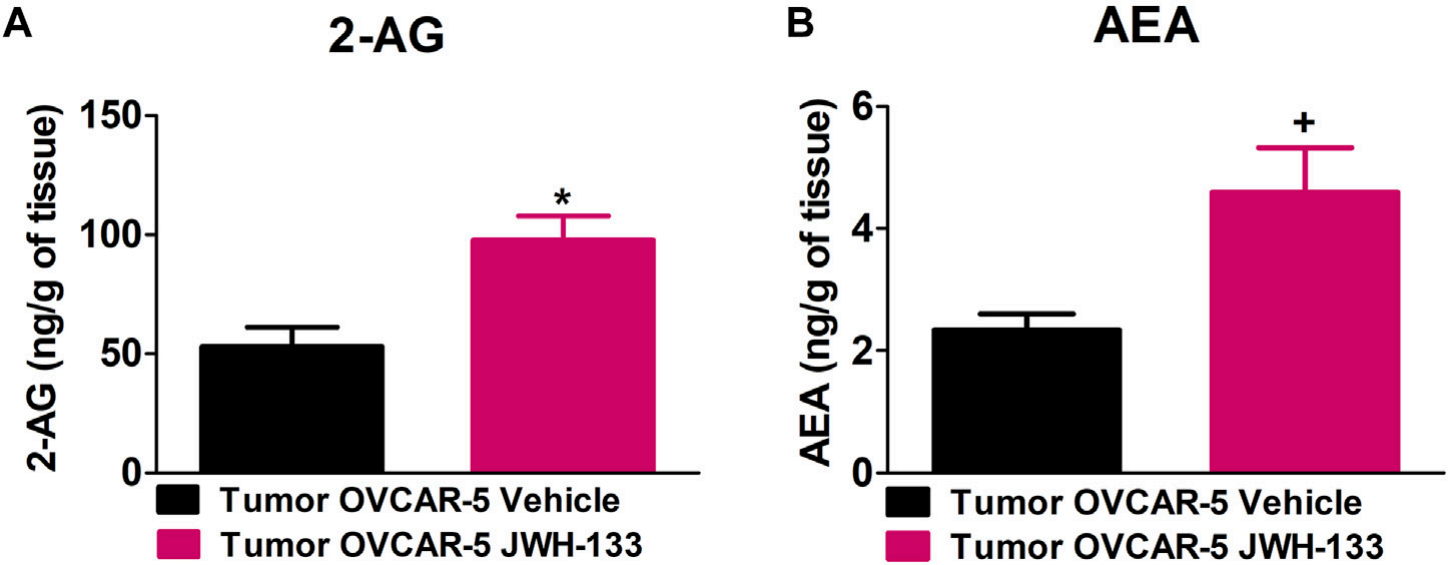
Increase in 2-arachidonoyl glycerol (2-AG) and anandamide (AEA) levels in the OVCAR-5 ectopic xenograft tumors of SCID-SHO female mice receiving chronic systemic administration of JWH-133. JWH-133 (1 mg/kg i.p.) chronic administration for 30 days starting on day 30 of tumor growth produced an increase in 2-AG **(A)** and AEA **(B)** levels in the OVCAR-5 ectopic xenograft tumors of SCID-SHO female mice relative to the vehicle treated group. Symbols represent the means ± SEM. (OVCAR-5 tumors in SCID-SHO female mice receiving vehicle, *n* = 5 SCID-SHO females; OVCAR-5 tumors in SCID-SHO female mice receiving JWH-133, *n* = 6 SCID-SHO females). *p < 0.0047 unpaired t-test (t_9_ = 3.295, one-tailed); +*p* < 0.0128 unpaired t-test (t_9_ = 2.669, one-tailed).

JWH-133 (1 mg/kg i.p.) chronic administration for 30 consecutive days starting on day 30 of tumor growth produced an increase in 2-AG (t_9_ = 3.295, *p* < 0.0047 unpaired t-test (one-tailed); Figure 5A) and AEA (t_9_ = 2.669, *p* < 0.0128 unpaired t-test (one-tailed); Figure 5B) levels in the OVCAR-5 ectopic xenograft tumors of SCID-SHO female mice relative to the vehicle treated group.

### 3.7 Increase in CB_2_ and TNFα but Decrease in GPER Protein Levels in the OVCAR-5 Ectopic Xenograft Tumors of SCID-SHO Female Mice Receiving Chronic Systemic administration of JWH-133

**FIGURE 6:**
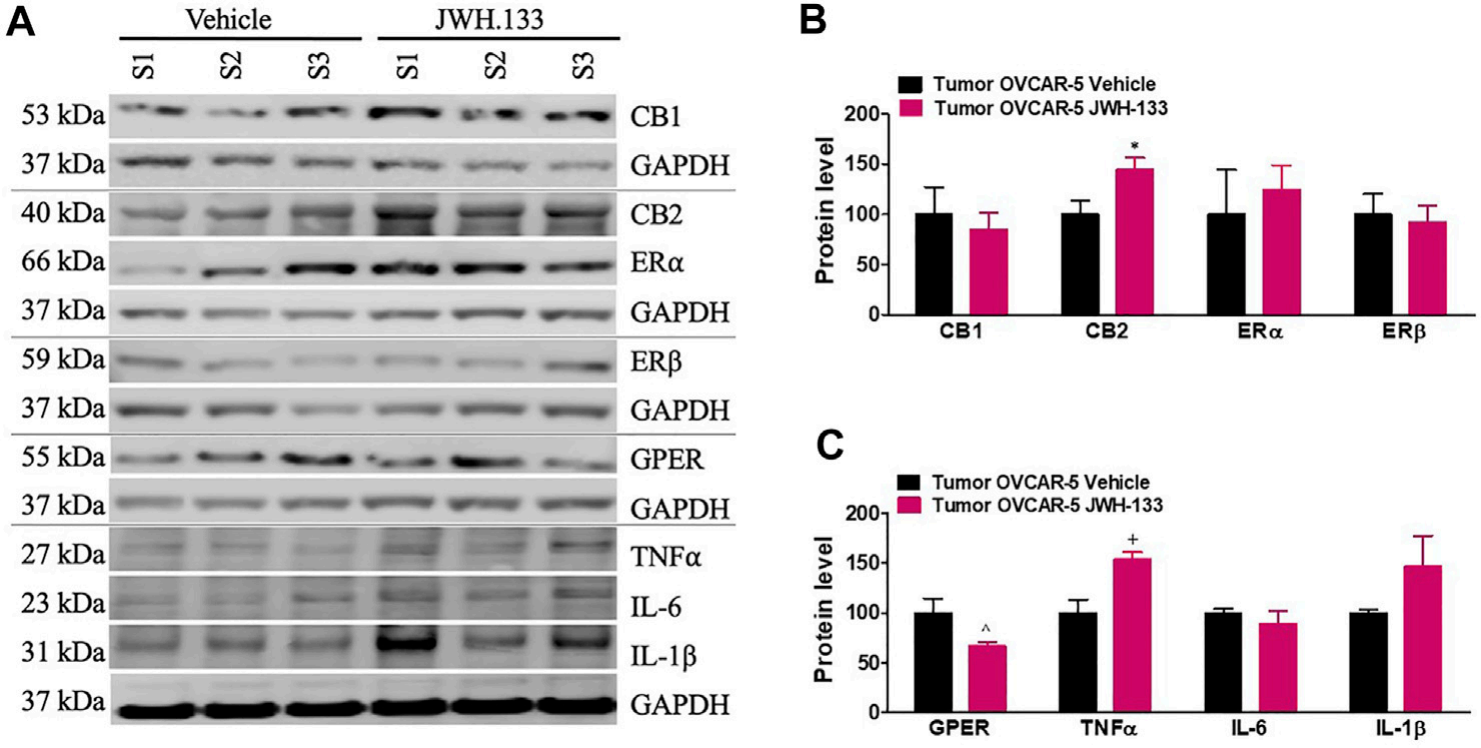
Increase in CB_2_ and TNFα but decrease in GPER protein levels in the OVCAR-5 ectopic xenograft tumors of SCID-SHO female mice receiving chronic systemic administration of JWH-133. JWH-133 (1 mg/kg i.p.) chronic administration for 30 days starting on day 30 of tumor growth produced an increase in CB_2_ **(A, B)** and TNFα **(A, C)** protein levels in the OVCAR-5 ectopic xenograft tumors of SCID-SHO female mice relative to the vehicle treated group. However, a decrease in GPER **(A, C)**) protein levels was in the OVCAR-5 ectopic xenograft tumors of SCID-SHO female mice receiving chronic administration of JWH-133 relative to vehicle group. Symbols represent the means ± SEM. (OVCAR-5 tumors in SCID-SHO female mice receiving vehicle, *n* = 3–6 SCID-SHO females; OVCAR-5 tumors in SCID-SHO female mice receiving JWH-133, *n* = 3–6 SCID-SHO females). *p < 0.033 unpaired t-test (t_4_ = 2.50, one-tailed); ^*p* < 0.0058 unpaired t-test (t_7_ = 3.387, one-tailed); +*p* < 0.0117 unpaired t-test (t_4_ = 3.568, one-tailed).

JWH-133 (1 mg/kg i.p.) chronic administration for 30 consecutive days starting on day 30 of tumor growth produced an increase in CB_2_ (t_4_ = 2.50, *p* < 0.0333 unpaired t-test (one-tailed); Figures 6A,B) and TNFα (t_4_ = 3.568, *p* < 0.0117 unpaired t-test (one-tailed); Figures 6A,C) protein levels in the OVCAR-5 ectopic xenograft tumors of SCID-SHO female mice relative to the vehicle treated group. However, a decrease in GPER (t_7_ = 3.387, *p* < 0.0058 unpaired t-test (one-tailed); Figures 6A,C)) protein levels was found in the OVCAR-5 ectopic xenograft tumors of SCID-SHO female mice receiving chronic administration of JWH-133 relative to vehicle treatment group. No changes in CB_1_ (t_10_ = 0.458, *p* = 0.3285 (ns) unpaired t-test (one-tailed)), ERα (t_10_ = 0.486, *p* = 0.3186 (ns) unpaired t-test (one-tailed)), ERβ (t_10_ = 0.295, *p* = 0.3871 (ns) unpaired t-test (one-tailed)), IL-6 (t_4_ = 0.839, *p* = 0.2241(ns) unpaired t-test (one-tailed)) and IL-1β (t_4_ = 1.520 *p* = 0.1015 (ns) unpaired t-test (one-tailed)) (Figures 6A–C) protein levels were found in the OVCAR-5 ectopic xenograft tumors of SCID-SHO female mice receiving chronic administration of JWH-133 in comparison to vehicle treatment group.

## 4 Discussion

Cannabinoids are used by cancer patients to alleviate nausea/vomiting and chemotherapy-induced peripheral neuropathy (Badowski, 2017; Blanton et al., 2019). Moreover, clinical evidence shows that the active component of cannabis, delta-9-tetrahydrocannabinol (∆^9^-THC) (Darkovska-Serafimovska et al., 2018; MacCallum and Russo, 2018) as well as pharmaceutical cannabinoids (Maida and Daeninck, 2016; Blake et al., 2017) can reduce chemotherapy-induced peripheral neuropathy in cancer patients. Increasing interest in the relationship between the endocannabinoid system and the anti-tumor actions of cannabinoids has been shown for different type of cancer (Guindon and Hohmann, 2011). However, majority of preclinical studies assessing the role of the endocannabinoid system on tumor growth have been conducted using *in vitro* experiments that inadequately mimic *in vivo* cancer progression (Guindon and Hohmann, 2011; Ladin et al., 2016). In our study, we demonstrate a significant increase in ectopic ovarian tumor growth following 30°days chronic administration of JWH-133. Furthermore, we also show that ovarian cancer tumor tissues chronically treated for 30 days with JWH-133 showed an increase in AEA and 2-AG and protein (CB_2_ and TNFα) levels and a decrease in GPER protein levels of ovarian cancer tumor tissue.

Recently, cannabinoids have gained increased attention for their effects on cancer growth (Guindon and Hohmann, 2011). Indeed, studies have focused their effort in using *in vivo* cancer models (Hanlon et al., 2016; Elbaz et al., 2017; Zhang et al., 2018). Previous work has evaluated the effect of CB_2_ (JWH-015, JWH-133) agonists on breast cancer models (Hanlon et al., 2016; Elbaz et al., 2017; Zhang et al., 2018). These studies have evaluated the effect of CB_2_ agonists on breast cancer models and shown a reduction in the size of breast cancer tumors (Hanlon et al., 2016; Elbaz et al., 2017; Zhang et al., 2018). At an early stage of tumor progression there is weaker angiogenesis processes (Lugano et al., 2019). Therefore, administration of CB_2_ agonist at this stage of tumor growth and for a short period of time will most likely not increase tumor growth. Indeed, it has been shown that CB_2_ agonist, JWH-015, given for 7 days can significantly attenuate bone remodeling, attenuate spontaneous pain and decrease primary tumor burden (Lozano-Ondoua et al., 2013). In our study, established ovarian tumors have stronger angiogenesis and we waited for 30°days of tumor growth before chronic administration of JWH-133 for a long period (30°days), we demonstrate a significant increase in ovarian cancer tumor growth due to increase levels of AEA and 2-AG.

Taken together, only a small number of preclinical studies have evaluated the impact of long-term treatment with CB_2_ agonists on tumor growth and cancer cell proliferation (Hanlon et al., 2016; Elbaz et al., 2017). Moreover, these studies evaluated the effects of CB_2_ agonists at an early stage of tumor growth (Hanlon et al., 2016; Zhang et al., 2018). However, studies assessing breast cancer progression that were using CB_2_ agonist administered within 7°days of tumor transplant have shown small tumor volumes under 100 mm^3^ using peritumoral (Elbaz et al., 2017) or systemic (Hanlon et al., 2016) administration.

There is an urgent need to improve our understanding of the long-term effects of cannabinoid-based therapies on tumor growth. Indeed, most studies have looked at the effect of cannabinoid compounds on tumor growth when the tumor starts growing. The possibility of enhanced tumor growth due to the side effects of analgesic compounds constitutes a significant problem that worsens the prognosis for cancer patients (Maley et al., 2017). Indeed, in cancer patients, cannabinoid compounds are administrated in the presence of already established and grown tumors. Therefore, we need to evaluate and study carefully the role played by the endocannabinoid system in cancer biology in preclinical studies using already established tumors before drawing general conclusion that cannabinoids reduce tumor growth in cancer patients (Cardoso et al., 2020). Using endogenous and exogenous cannabinoids in cancer patients for their anti-cancer properties needs to be considered not only in terms of their potential anti-cancer effects but keeping in mind their wide range of effects (cell death, regulation of angiogenesis and invasion as well as anticancer immunity) that cannabinoid compounds can generate (Cardoso et al., 2020; Moreno et al., 2020; McHann et al., 2021). The role of cannabinoid compounds in the modulation of tumor growth is directly linked to hormones, tumor types and endocannabinoids involved in a specific type of cancer (Moreno et al., 2020; Aylar and Hassan, 2021; McHann et al., 2021). Furthermore, only few pilot clinical trials have evaluated the effect of cannabinoid compounds in patients (Hinz and Ramer, 2019). Indeed, the classic plant-derived cannabinoid delta-9-tetrahydrocannabinol (∆^9^-THC) acts as a partial agonist at cannabinoid receptors (CB_1_ and CB_2_) (Pertwee, 2008), and it was demonstrated to be safe in glioblastoma patients (Guzmán et al., 2006). The nuclear protein Ki67 (pKi67) is an established marker for cancer diagnosis and treatment, and its expression is strongly associated with tumor cell proliferation and growth (Li et al., 2015). Moreover, the treatment of human patient-derived glioblastoma cells with ∆^9^-THC has been shown to reduce the number of Ki67-labelled cells, through the orphan cannabinoid receptor GPR55 (Kolbe et al., 2021). Cannabidiol, another plant-derived cannabinoid (McPartland and Russo, 2001), has negligible affinity for the cloned CB_1_ and CB_2_ receptors (Showalter et al., 1996). Cannabidiol treatment also inhibits the proliferation of human glioblastoma cell lines (Marcu et al., 2010). Moreover, it has been demonstrated that cannabidiol enhances the inhibitory effects of ∆^9^-THC on cell proliferation and survival of human glioblastoma cells, inducing cell cycle arrest and apoptosis (Marcu et al., 2010).

In our study, we evaluated the effect of chronic administration of CB_2_ (JHW-133) agonist in ovarian cancer in 30°days ovarian tumors. We wanted to evaluate the effect of chronic administration of JWH-133 once a tumor is established for 1 month (30°days). We demonstrated a significant increase in tumor growth following JWH-133 administration relative to vehicle treated group. This increase in ectopic ovarian tumor growth was associated with increase anandamide and 2-AG in ovarian cancer tumor tissue chronically treated with JWH-133 for 30°days. Interestingly, it has been demonstrated that monoacylglycerol lipase (MAGL) activity is involve in inflammation (Guindon et al., 2011), but has also been implicated to play a pathophysiological role in cancer (Nomura et al., 2010; Deng and Li, 2020). Indeed, it has been demonstrated that MAGL activity is highly elevated in multiple type of aggressive human cancer cells such as ovarian, breast and melanoma cancer cells (Nomura et al., 2010). It was demonstrated that MAGL is involved in cellular processes (cellular growth, survival, migration and invasion) and promotes cancer aggressiveness by providing a pool of free fatty acids for oncogenic signaling of lipid synthesis (Nomura et al., 2010). It was demonstrated that MAGL inhibition can impaired prostate cancer aggressiveness (Nomura et al., 2010). Moreover, MAGL inhibition has been also associated with alleviation of chemotherapy-induced peripheral neuropathy (Guindon and Hohmann, 2011; Guindon et al., 2013). Our study demonstrates that chronic treatment of JWH-133 for 30 consecutive days in ovarian cancer tumors already developed (30°days) increases levels of AEA and 2-AG and is associated with an increase in tumor size relative to vehicle treated group.

Furthermore, it was demonstrated, in an ectopic xenograft colon cancer model, that administration of CB_2_ (JWH-133 and HU-308) agonists promoted cancer progression (Martínez-Martínez et al., 2016). The mechanism involved for this increase in colon cancer progression was via serine/threonine protein kinase (AKT)/Glycogen synthase kinase 3 (GSK_3_) beta signaling pathway (Martínez-Martínez et al., 2016). In our study, we find an increase in CB_2_ and TNFα and decrease in GPER protein levels. Further studies are needed to better understand the specific pathway and mechanisms involved in the increase in ectopic ovarian cancer growth and protein levels of CB_2_ and TNFα following chronic administration of CB_2_ (JWH-133) agonist.

Interestingly, our findings suggest that chronic administration of CB_2_ agonist should most likely be combined with FAAH and/or MAGL inhibitors to prevent the generation of a pool of free fatty acid leading to increase in tumor growth and endocannabinoid (AEA, 2-AG) levels in ovarian cancer. Moreover, recent studies have shown that combination of JWH-133 and light irradiation inhibit tumor growth in triple negative breast cancer (Zhang et al., 2018). Therefore, the combination of CB_2_ agonist with FAAH and/or MAGL inhibitors with light irradiation could potentially and significantly reduce the aggressiveness of breast and/or ovarian cancer.

## 5 Conclusion

In this study, we demonstrate for the first time, a significant increase in ectopic ovarian tumor growth following 30°days chronic administration of JWH-133. Furthermore, ovarian cancer tumor tissues chronically (30°days) treated with JWH-133 showed an increase in AEA and 2-AG and protein (CB_2_ and TNFα) levels. However, we observed a decrease in GPER protein levels of ovarian cancer tumor tissue. Cannabinoids are increasingly used in cancer patients to alleviate nausea/vomiting and chemotherapy-induced peripheral neuropathy (Blanton et al., 2019) therefore a clear understanding of their effect on tumor growth following chronic administration in *already established tumors* is essential. Our study emphasizes the importance of studying the impact of cannabinoid compounds on already established tumors to improve our understanding and the long-term effects of cannabinoid-based therapies on tumor growth and, therefore better address clinical needs in cancer patients.

## Data Availability

The original contributions presented in the study are included in the article/Supplementary Material, further inquiries can be directed to the corresponding authors.

## Abbreviations

2-AG, 2-arachidonoyl glycerol; ∆9-THC, delta-9-tetrahydrocannabinol; AEA, anandamide; AKT: serine—threonine kinase, which is also known as protein kinase B; ANOVA: analysis of variance; BL: baseline; CB1: cannabinoid receptor type 1; CB2: cannabinoid receptor type 2; Erα, estrogen receptor alpha; Erβ, estrogen receptor beta; GADPH, glyceraldehyde-3-phosphate dehydrogenase; GSK3: glycogen synthase kinase-3β; GPER, G protein-coupled estrogen receptor; IL-1β, interleukin 1 beta; IL-6, interleukin 6; LC/MS, liquid chromatography-mass spectrometry; MAGL, monoacylglycerol lipase; ns, not significant; TNFα, tumor necrosis factor alpha.

## References

[B1] Aylar A. Hassan M. (2021). The Molecular Targets of Cannabinoids in the Treatment of Cancer and Inflammation. Curr. Pharm. Des. 27 (25), 2881–2892. 10.2174/1381612827666210426092847 33902407

[B2] Badowski M. E. (2017). A Review of Oral Cannabinoids and Medical Marijuana for the Treatment of Chemotherapy-Induced Nausea and Vomiting: a Focus on Pharmacokinetic Variability and Pharmacodynamics. Cancer Chemother. Pharmacol. 80 (3), 441–449. 10.1007/s00280-017-3387-5 28780725 PMC5573753

[B3] Blake A. Wan B. A. Malek L. DeAngelis C. Diaz P. Lao N. (2017). A Selective Review of Medical Cannabis in Cancer Pain Management. Ann. Palliat. Med. 6 (Suppl. 2), S215–S22. 10.21037/apm.2017.08.05 28866904

[B4] Blankman J. L. Simon G. M. Cravatt B. F. (2007). A Comprehensive Profile of Brain Enzymes that Hydrolyze the Endocannabinoid 2-arachidonoylglycerol. Chem. Biol. 14 (12), 1347–1356. 10.1016/j.chembiol.2007.11.006 18096503 PMC2692834

[B5] Blanton H. L. Brelsfoard J. DeTurk N. Pruitt K. Narasimhan M. Morgan D. J. (2019). Cannabinoids: Current and Future Options to Treat Chronic and Chemotherapy-Induced Neuropathic Pain. Drugs 79 (9), 969–995. 10.1007/s40265-019-01132-x 31127530 PMC8310464

[B6] Booth M. (2003). Cannabis: A History. New York, NY: Picador, St. Martin’s Press.

[B7] Bradshaw H. B. Rimmerman N. Krey J. F. Walker J. M. (2006). Sex and Hormonal Cycle Differences in Rat Brain Levels of Pain-Related Cannabimimetic Lipid Mediators. Am. J. Physiol. Regul. Integr. Comp. Physiol. 291 (2), R349–R358. 10.1152/ajpregu.00933.2005 16556899

[B8] Cardoso F. Paluch-Shimon S. Senkus E. Curigliano G. Aapro M. S. André F. (2020). 5th ESO-ESMO International Consensus Guidelines for Advanced Breast Cancer (ABC 5). Ann. Oncol. 31 (12), 1623–1649. 10.1016/j.annonc.2020.09.010 32979513 PMC7510449

[B9] Castro-Piedras I. Sharma M. Brelsfoard J. Vartak D. Martinez E. G. Rivera C. (2021). Nuclear Dishevelled Targets Gene Regulatory Regions and Promotes Tumor Growth. EMBO Rep. 22 (6), e50600. 10.15252/embr.202050600 33860601 PMC8183407

[B10] Cravatt B. F. Giang D. K. Mayfield S. P. Boger D. L. Lerner R. A. Gilula N. B. (1996). Molecular Characterization of an Enzyme that Degrades Neuromodulatory Fatty-Acid Amides. Nature 384 (6604), 83–87. 10.1038/384083a0 8900284

[B11] Darkovska-Serafimovska M. Serafimovska T. Arsova-Sarafinovska Z. Stefanoski S. Keskovski Z. Balkanov T. (2018). Pharmacotherapeutic Considerations for Use of Cannabinoids to Relieve Pain in Patients with Malignant Diseases. J. Pain Res. 11, 837–842. 10.2147/JPR.S160556 29719417 PMC5922297

[B12] Deng H. Li W. (2020). Monoacylglycerol Lipase Inhibitors: Modulators for Lipid Metabolism in Cancer Malignancy, Neurological and Metabolic Disorders. Acta Pharm. Sin B 10 (4), 582–602. 10.1016/j.apsb.2019.10.006 32322464 PMC7161712

[B13] Devane W. A. Dysarz F. A. 3rd Johnson M. R. Melvin L. S. Howlett A. C. (1988). Determination and Characterization of a Cannabinoid Receptor in Rat Brain. Mol. Pharmacol. 34 (5), 605–613. 2848184

[B14] Devane W. A. Hanus L. Breuer A. Pertwee R. G. Stevenson L. A. Griffin G. (1992). Isolation and Structure of a Brain Constituent that Binds to the Cannabinoid Receptor. Science 258 (5090), 1946–1949. 10.1126/science.1470919 1470919

[B15] Dinh T. P. Carpenter D. Leslie F. M. Freund T. F. Katona I. Sensi S. L. (2002). Brain Monoglyceride Lipase Participating in Endocannabinoid Inactivation. Proc. Natl. Acad. Sci. U S A. 99 (16), 10819–10824. 10.1073/pnas.152334899 12136125 PMC125056

[B16] Elbaz M. Ahirwar D. Ravi J. Nasser M. W. Ganju R. K. (2017). Novel Role of Cannabinoid Receptor 2 in Inhibiting EGF/EGFR and IGF-I/IGF-IR Pathways in Breast Cancer. Oncotarget 8 (18), 29668–29678. 10.18632/oncotarget.9408 27213582 PMC5444694

[B17] Goparaju S. K. Ueda N. Taniguchi K. Yamamoto S. (1999). Enzymes of Porcine Brain Hydrolyzing 2-arachidonoylglycerol, an Endogenous Ligand of Cannabinoid Receptors. Biochem. Pharmacol. 57 (4), 417–423. 10.1016/s0006-2952(98)00314-1 9933030

[B18] Guindon J. Guijarro A. Piomelli D. Hohmann A. G. (2011). Peripheral Antinociceptive Effects of Inhibitors of Monoacylglycerol Lipase in a Rat Model of Inflammatory Pain. Br. J. Pharmacol. 163 (7), 1464–1478. 10.1111/j.1476-5381.2010.01192.x 21198549 PMC3165956

[B19] Guindon J. Hohmann A. G. (2011). The Endocannabinoid System and Cancer: Therapeutic Implication. Br. J. Pharmacol. 163 (7), 1447–1463. 10.1111/j.1476-5381.2011.01327.x 21410463 PMC3165955

[B20] Guindon J. Lai Y. Takacs S. M. Bradshaw H. B. Hohmann A. G. (2013). Alterations in Endocannabinoid Tone Following Chemotherapy-Induced Peripheral Neuropathy: Effects of Endocannabinoid Deactivation Inhibitors Targeting Fatty-Acid Amide Hydrolase and Monoacylglycerol Lipase in Comparison to Reference Analgesics Following Cisplatin Treatment. Pharmacol. Res. 67 (1), 94–109. 10.1016/j.phrs.2012.10.013 23127915 PMC3525790

[B21] Guzmán M. Duarte M. J. Blázquez C. Ravina J. Rosa M. C. Galve-Roperh I. (2006). A Pilot Clinical Study of Delta9-tetrahydrocannabinol in Patients with Recurrent Glioblastoma Multiforme. Br. J. Cancer 95 (2), 197–203. 10.1038/sj.bjc.6603236 16804518 PMC2360617

[B22] Gaoni Y. Mechoulam R. (1964). Isolation, Structure, and Partial Synthesis of an Active Constituent of Hashish. J. Am. Chem. Soc. 86 (8), 1646–1647.

[B23] Hanlon K. E. Lozano-Ondoua A. N. Umaretiya P. J. Symons-Liguori A. M. Chandramouli A. Moy J. K. (2016). Modulation of Breast Cancer Cell Viability by a Cannabinoid Receptor 2 Agonist, JWH-015, Is Calcium Dependent. Breast Cancer (Dove Med. Press. 8, 59–71. 10.2147/BCTT.S100393 27186076 PMC4847606

[B24] Hinz B. Ramer R. (2019). Anti-tumour Actions of Cannabinoids. Br. J. Pharmacol. 176 (10), 1384–1394. 10.1111/bph.14426 30019449 PMC6487602

[B25] Kolbe M. R. Hohmann T. Hohmann U. Ghadban C. Mackie K. Zöller C. (2021). THC Reduces Ki67-Immunoreactive Cells Derived from Human Primary Glioblastoma in a GPR55-dependent Manner. Cancers (Basel) 13 (5), 1064. 10.3390/cancers13051064 33802282 PMC7959141

[B26] Kramer J. L. (2015). Medical Marijuana for Cancer. CA Cancer J. Clin. 65 (2), 109–122. 10.3322/caac.21260 25503438

[B27] Ladin D. A. Soliman E. Griffin L. Van Dross R. (2016). Preclinical and Clinical Assessment of Cannabinoids as Anti-cancer Agents. Front. Pharmacol. 7, 361. 10.3389/fphar.2016.00361 27774065 PMC5054289

[B28] Li L. T. Jiang G. Chen Q. Zheng J. N. (2015). Ki67 Is a Promising Molecular Target in the Diagnosis of Cancer (Review). Mol. Med. Rep. 11 (3), 1566–1572. 10.3892/mmr.2014.2914 25384676

[B29] Lozano-Ondoua A. N. Hanlon K. E. Symons-Liguori A. M. Largent-Milnes T. M. Havelin J. J. Ferland H. L. 3rd (2013). Disease Modification of Breast Cancer-Induced Bone Remodeling by Cannabinoid 2 Receptor Agonists. J. Bone Miner Res. 28 (1), 92–107. 10.1002/jbmr.1732 22903605 PMC4745976

[B30] Luca T. Di Benedetto G. Scuderi M. R. Palumbo M. Clementi S. Bernardini R. (2009). The CB1/CB2 Receptor Agonist WIN-55,212-2 Reduces Viability of Human Kaposi's Sarcoma Cells *In Vitro* . Eur. J. Pharmacol. 616 (1-3), 16–21. 10.1016/j.ejphar.2009.06.004 19539619

[B31] MacCallum C. A. Russo E. B. (2018). Practical Considerations in Medical Cannabis Administration and Dosing. Eur. J. Intern. Med. 49, 12–19. 10.1016/j.ejim.2018.01.004 29307505

[B32] Maida V. Daeninck P. J. (2016). A User's Guide to Cannabinoid Therapies in Oncology. Curr. Oncol. 23 (6), 398–406. 10.3747/co.23.3487 28050136 PMC5176373

[B33] Maley C. C. Aktipis A. Graham T. A. Sottoriva A. Boddy A. M. Janiszewska M. (2017). Classifying the Evolutionary and Ecological Features of Neoplasms. Nat. Rev. Cancer 17 (10), 605–619. 10.1038/nrc.2017.69 28912577 PMC5811185

[B34] Marcu J. P. Christian R. T. Lau D. Zielinski A. J. Horowitz M. P. Lee J. (2010). Cannabidiol Enhances the Inhibitory Effects of delta9-tetrahydrocannabinol on Human Glioblastoma Cell Proliferation and Survival. Mol. Cancer Ther. 9 (1), 180–189. 10.1158/1535-7163.MCT-09-0407 20053780 PMC2806496

[B35] Martínez-Martínez E. Martín-Ruiz A. Martín P. Calvo V. Provencio M. García J. M. (2016). CB2 Cannabinoid Receptor Activation Promotes colon Cancer Progression via AKT/GSK3β Signaling Pathway. Oncotarget 7 (42), 68781–68791. 10.18632/oncotarget.11968 27634891 PMC5356589

[B36] Maureen A. Harrison I. F. R. (1997). General Techniques of Cell Culture. London: Cambridge University Press.

[B37] McHann M. C. Blanton H. L. Guindon J. (2021). Role of Sex Hormones in Modulating Breast and Ovarian Cancer Associated Pain. Mol. Cel Endocrinol 533, 111320. 10.1016/j.mce.2021.111320 PMC8263503 34033890

[B38] McPartland J. M. Russo E. B. (2001). Cannabis and Cannabis Extracts. J. Cannabis Ther. 1 (3-4), 103–132. 10.1300/j175v01n03_08

[B39] Mechoulam R. Ben-Shabat S. Hanus L. Ligumsky M. Kaminski N. E. Schatz A. R. (1995). Identification of an Endogenous 2-monoglyceride, Present in Canine Gut, that Binds to Cannabinoid Receptors. Biochem. Pharmacol. 50 (1), 83–90. 10.1016/0006-2952(95)00109-d 7605349

[B40] Moreno E. Cavic M. Krivokuca A. Canela E. I. (2020). The Interplay between Cancer Biology and the Endocannabinoid System-Significance for Cancer Risk, Prognosis and Response to Treatment. Cancers (Basel) 12 (11), 3275. 10.3390/cancers12113275 PMC7694406 33167409

[B41] Munro S. Thomas K. L. Abu-Shaar M. (1993). Molecular Characterization of a Peripheral Receptor for Cannabinoids. Nature 365 (6441), 61–65. 10.1038/365061a0 7689702

[B42] National Research Council DoEaLS, Institute for Laboratory Animal Research (2011). Guide for the Care and Use of Laboratory Animals: Eighth Edition 2011. Washington, DC: National Academies Press (US).

[B43] Nomura D. K. Long J. Z. Niessen S. Hoover H. S. Ng S. W. Cravatt B. F. (2010). Monoacylglycerol Lipase Regulates a Fatty Acid Network that Promotes Cancer Pathogenesis. Cell 140 (1), 49–61. 10.1016/j.cell.2009.11.027 20079333 PMC2885975

[B44] Parker L. A. Rock E. M. Limebeer C. L. (2011). Regulation of Nausea and Vomiting by Cannabinoids. Br. J. Pharmacol. 163 (7), 1411–1422. 10.1111/j.1476-5381.2010.01176.x 21175589 PMC3165951

[B45] Pertwee R. G. (2008). The Diverse CB1 and CB2 Receptor Pharmacology of Three Plant Cannabinoids: delta9-tetrahydrocannabinol, Cannabidiol and delta9-tetrahydrocannabivarin. Br. J. Pharmacol. 153 (2), 199–215. 10.1038/sj.bjp.0707442 17828291 PMC2219532

[B46] Scuderi M. R. Cantarella G. Scollo M. Lempereur L. Palumbo M. Saccani-Jotti G. (2011). The Antimitogenic Effect of the Cannabinoid Receptor Agonist WIN55212-2 on Human Melanoma Cells Is Mediated by the Membrane Lipid Raft. Cancer Lett. 310 (2), 240–249. 10.1016/j.canlet.2011.07.008 21807457

[B47] Showalter V. M. Compton D. R. Martin B. R. Abood M. E. (1996). Evaluation of Binding in a Transfected Cell Line Expressing a Peripheral Cannabinoid Receptor (CB2): Identification of Cannabinoid Receptor Subtype Selective Ligands. J. Pharmacol. Exp. Ther. 278 (3), 989–999. 8819477

[B48] Zhang J. Zhang S. Liu Y. Su M. Ling X. Liu F. (2018). Combined CB2 Receptor Agonist and Photodynamic Therapy Synergistically Inhibit Tumor Growth in Triple Negative Breast Cancer. Photodiagnosis Photodyn Ther. 24, 185–191. 10.1016/j.pdpdt.2018.09.006 30240926 PMC6289793

